# Free Open Access Medical Education (FOAM) in Emergency Medicine: The Global Distribution of Users in 2016

**DOI:** 10.5811/westjem.2018.3.36825

**Published:** 2018-04-05

**Authors:** Taylor W. Burkholder, Jennifer W. Bellows, Renee A. King

**Affiliations:** * University of Colorado, School of Medicine, Department of Emergency Medicine, Aurora, Colorado; † Denver Health & Hospital Authority, Department of Emergency Medicine, Denver, Colorado

## Abstract

**Introduction:**

Free open-access medical education (FOAM) is a collection of interactive online medical education resources—free and accessible to students, physicians and other learners. This novel approach to medical education has the potential to reach learners across the globe; however, the extent of its global uptake is unknown.

**Methods:**

This descriptive report evaluates the 2016 web analytics data from a convenience sample of FOAM blogs and websites with a focus on emergency medicine (EM) and critical care. The number of times a site was accessed, or “sessions”, was categorized by country of access, cross-referenced with World Bank data for population and income level, and then analyzed using simple descriptive statistics and geographic mapping.

**Results:**

We analyzed 12 FOAM blogs published from six countries, with a total reported volume of approximately 18.7 million sessions worldwide in 2016. High-income countries accounted for 73.7% of population-weighted FOAM blog and website sessions in 2016, while upper-middle income countries, lower-middle income countries and low-income countries accounted for 17.5%, 8.5% and 0.3%, respectively.

**Conclusion:**

FOAM, while largely used in high-income countries, is used in low- and middle-income countries as well. The potential to provide free, online training resources for EM in places where formal training is limited is significant and thus is prime for further investigation.

## INTRODUCTION

Free open-access medical education (FOAM) is a collection of interactive online medical education resources—free and accessible to students, physicians, nurses, paramedics and other learners.^1^ FOAM uses multiple online platforms such as blogs, podcasts, tweets, videos and other web-based media to form a community that shares ideas and accelerates the translation of research into clinical practice.^1^–^3^ Physicians in emergency medicine (EM) and critical care have been leaders in the trend to rapidly increase the number of online resources that share FOAM content, and recently there have been calls to formally integrate online learning into residency education in the United States.^4^,^5^

Formal training in EM is lacking in many low- and middle-income countries (LMICs) but must be prioritized in order to reach key development priorities for emergency care systems.^6^,^7^ FOAM has the potential to fill certain gaps in EM training resources in LMICs. The current content of FOAM represents a diverse array of learning resources from core emergency care basics to cutting-edge techniques such as extracorporeal membrane oxygenation. Although the latter is unlikely to be relevant in low-resource contexts, there is potential for content to be customized to the resources and cultural context of a country, as opposed to textbooks written predominantly for high-resourced settings. However, awareness of FOAM resources may in fact be lowest in those LMIC settings where formal resources (e.g. textbooks, lecturers, instructors, simulations) are least available.^8^ This descriptive report assesses the global uptake of FOAM via the geographical distribution of blog and website users in 2016.

## METHODS

A convenience sample of popular FOAM blogs and websites—known to the authors or identified via a Google search using the term “emergency medicine FOAM”—were approached for inclusion via email inquiries. We identified additional sites by referral of the site administrators that responded to emails. Sites were included if they were free, fully accessible, had actively published new content in 2016, and specifically addressed mainstream topics in EM and critical care. We excluded sites if they solely produced niche content that is less applicable to the wider global audience (e.g. emergency subspecialties such as wilderness medicine). Web analytics data for all sites were collected via Google Analytics to ascertain the location of de-identified users accessing the blog or website in the calendar year 2016. No individual Internet protocol addresses were collected, nor are they available from the version of Google Analytics used. We grouped the number of sessions—or unique interactions between a user and the site—by country of access.

For each country, we calculated a cumulative number of sessions from all websites and blogs, which was then cross-referenced with World Bank data for population and income level. To account for large differences in population sizes between countries (and therefore large differences in potential FOAM users), population-weighted session counts (sessions per million people) were calculated by dividing the gross number of sessions by the 2016 World Bank population figure for each country, then multiplied by one million.

We then grouped countries as high income, upper-middle income, lower-middle income and low income by 2016 World Bank classification. Gross session counts and population-weighted session counts for each economic stratum were again calculated in the manner described above.

All data were aggregated in Microsoft Excel (v.14.5.5, Redmond, WA) and analyzed via simple descriptive statistics. We mapped cumulative and population-weighted session counts for visualization of the global distribution using Infogram (Infogram Software Inc., San Francisco, CA).

## RESULTS

We included 12 FOAM blogs and websites from six countries for analysis (Table 1). The majority of sites were published in English, while one site (MDU Chile) was published in Spanish and another (FOAM EM) aggregated blog postings from multiple languages. The combined reported annual sessions of these FOAM sites totaled approximately 18.7 million sessions worldwide in 2016. The number of unique countries accessing each site ranged from 82 to 209.

**Population Health Research Capsule**

What do we already know about this issue?

Free open-access medical education (FOAM) is a novel approach to education that has potential to reach emergency medicine (EM) learners worldwide.

What was the research question?

To what extent is FOAM being used by EM learners around the globe?

What was the major finding of the study?

FOAM is mostly used in high-income countries, but there are notable users in several middle-income countries.

How does this improve population health?

FOAM is prime for further research regarding its ability to train EM providers around the world.

**Figure 1:**
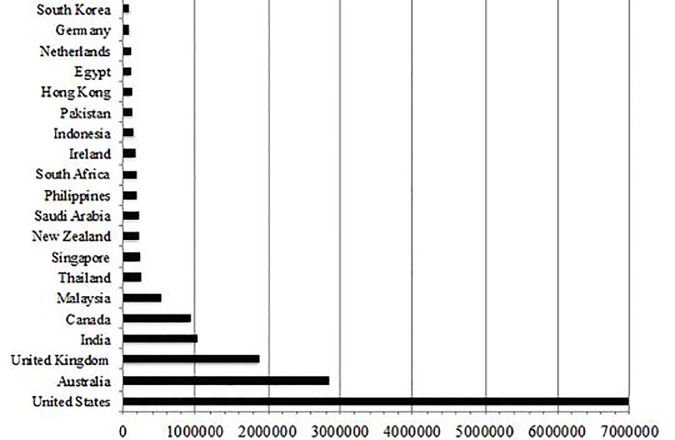
Gross annual sessions from FOAM users in the top 20 countries, 2016. *FOAM*, Free Open Access Medical education.

**Figure 2:**
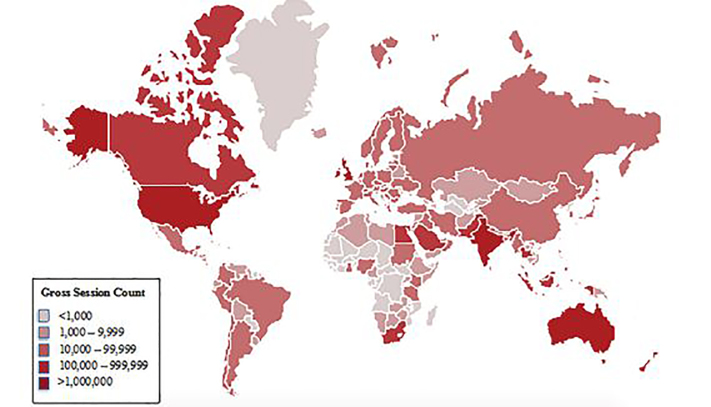
Global FOAM distribution- Gross annual session counts by country, 2016. *FOAM*, Free Open Access Medical education.

**Figure 3:**
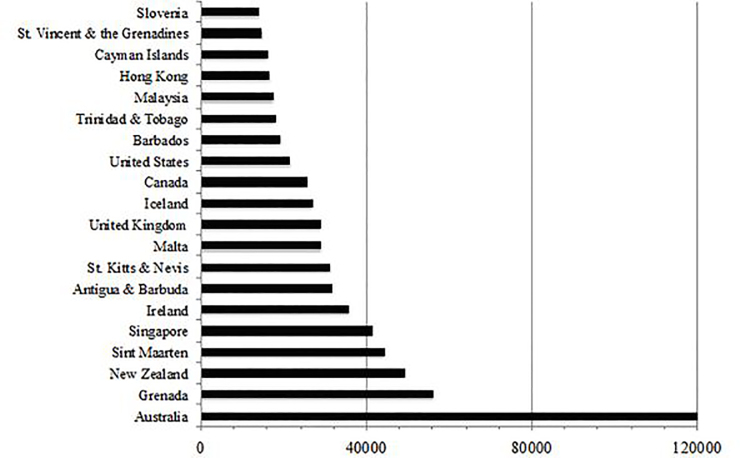
Population-weighted session counts from FOAM users in the top 20 countries, 2016 (per million people). *FOAM*, Free Open Access Medical education.

**Figure 4:**
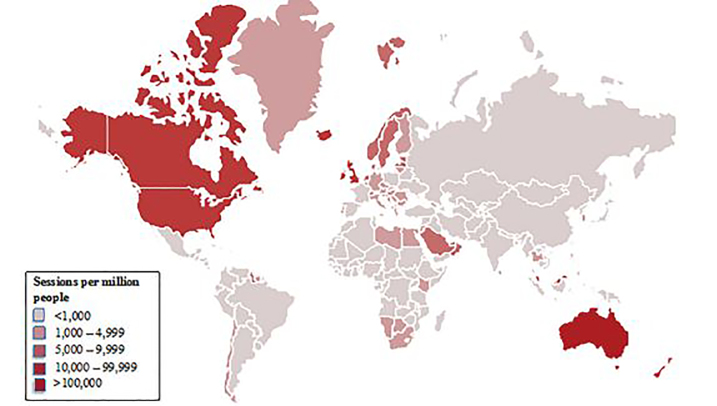
Global FOAM distribution- Population-weighted session counts by country, 2016. *FOAM*, Free Open Access Medical education.

The 20 countries with the highest gross annual sessions in 2016 are listed in Figure 1. The United States, Australia, the United Kingdom and India had cumulative session counts greater than one million. Figure 2 maps the global distribution of users by gross annual session counts. Figure 3 shows the population-weighted session counts for the 20 countries with the most FOAM activity, and Figure 4 maps the global distribution of users by population-weighted session counts.

When population-weighted session counts were grouped by World Bank income classification, we noted diminishing usage of FOAM blogs and websites as income level decreased. High-income countries accounted for 73.7% of population-weighted FOAM blog and website sessions in 2016, while upper-middle income countries, lower-middle countries and low-income countries accounted for 17.5%, 8.5% and 0.3%, respectively (Table 2).

## DISCUSSION

The majority of users of FOAM blogs and websites are concentrated in a small number of countries, many of which are also the primary producers of FOAM content such as the U.S., Australia, Canada, and the United Kingdom. Conversely, there are large gaps in FOAM use in many regions of South America, central Africa, and Asia where language and economic development might present challenges to access and use. Other potential barriers to FOAM use in these regions include web accessibility and speed, device availability, censorship, and lack of awareness.

Despite the majority of FOAM users clustering in high-income countries, there is a notable signal of user activity in several middle-income countries, which suggests a potential audience for FOAM content beyond the current high-income users. For example, South Africa is an upper-middle income country that accounted for 195,070 of the gross FOAM sessions in 2016. The country is also home to several graduate EM training programs dating back to 2001, which may explain the relative increase in FOAM users as compared to other LMICs.^9^

These findings, although a single snapshot of FOAM usage, represent a baseline index that can be used in future years to assess the growth and penetration of FOAM resources into LMICs. Since FOAM users have begun to emerge in many LMICs, we suggest that FOAM content creators consider developing a subset of FOAM that is particularly relevant to resource-limited contexts. Additionally, we encourage a partnership between experienced FOAM creators with clinicians and educators in LMICs that have an interest in developing their own FOAM content. This type of mentorship will provide a vehicle for clinicians in LMICs to publish educational materials and to diversify the current scope of FOAM.

## LIMITATIONS

There are several limitations to the generalizability of our findings. Due to the lack of standardized cataloguing of FOAM resources, we were unaware of a truly systematic method of sampling all FOAM sites. Instead, our convenience sample was limited to those sites that were already known to the authors, readily identified as top hits by a Google search, or referred by other site administrators. In many cases there was no response to email inquiries; thus, no website data could be obtained. We exclusively sampled FOAM blogs and websites related to EM and critical care. Our results may not be fully representative of other platforms of FOAM, such as podcasts or videos, or FOAM content tailored to other medical and surgical specialties. Ten out of the 12 sites were published in English. Unless this language allocation is truly representative of the published FOAM content, our findings likely under-report the number of sessions from non-English speaking countries.

Although we posit that the number of sessions originating from a particular country approximates the number of users in that country, this may only loosely estimate the true distribution of users. The advantage of this method is that it takes into account both the number of users and their degree of activity (number of unique visits to a site) over the course of the year. However, we were unable to determine if a smaller core of very active users gain more from FOAM resources than a larger audience of infrequent users. Our method also assumes that a negligible number of FOAM users are accessing virtual private networks, which would falsely lower the session counts from a particular country.

Ideally, a weighted session count would be cross-referenced by the number of healthcare providers (i.e., end users of FOAM) in a given country. However, these data were not readily available for most countries, so session counts were weighted by country population size instead. In many island nations such as Grenada the population size is small, but weighted session counts may be easily skewed by the presence of medical schools that draw from the international community.

Finally, this study does not answer important questions about barriers to awareness and use of FOAM in LMICs. Further investigation is needed to understand the potential impact of FOAM on EM training, the availability of the Internet and web-enabled devices required to access FOAM, the growth of FOAM over time, and the applicability of FOAM content to practicing in low-resource settings. A needs assessment of learners in LMICs would be helpful to understand the gaps in educational resources and whether FOAM has the potential to fill those gaps.

## CONCLUSION

Our findings suggest that FOAM is largely being used in a select number of high-income countries. However, there are significant numbers of users in middle-income countries as well. The potential to provide free, online training resources for emergency medicine in places where formal training is limited is prime for further investigation.

## Figures and Tables

**Table 1:** Description of FOAM blogs and websites included for analysis, 2016.

Site	Country of origin	Language	Annual sessions	Number of countries accessing
Life in the fast lane	Australia	English	17,436,575	209
ALiEM	USA	English	568,521	196
Pediatric EM morsels	USA	English	245,264	187
FOAM EM	UK	Multiple	196,628	187
ER cast	USA	English	119,388	170
Intensive blog	Australia	English	76,026	169
Broome docs	Australia	English	57,401	170
SOC MOB	Canada	English	44,097	158
EM tutorials	New Zealand	English	39,818	168
SCGH ED	Australia	English	33,969	156
Manu Et corde	Canada	English	27,606	156
Blunt dissection	Australia	English	16,628	139
MDU Chile	Chile	Spanish	10,941	80
EM/IM doc	USA	English	4,164	82

*FOAM*, free open access medical education; *ALiEM*, Academic Life in Emergency Medicine; *EM*, emergency medicine; *ER*, emergency room; *SOC MOB*, standing on the corner, minding my own business; *SCGH ED*, Sir Charles Gairdner Hospital Emergency Department; *MDU*, Medicine de Urgencia; *IM*, Internal Medicine; *USA*, United States of America; *UK*, United Kingdom.

**Table 2:** Distribution of FOAM sessions by World Bank income level, 2016.

Income level*	Total sessions	% of Total sessions	Sessions per million people	% of Sessions per million people
High-income	14,067,663	75.30%	806,043	73.72%
Upper-middle income	1,604,520	8.59%	190,835	17.45%
Lower-middle income	2,933,755	15.70%	93,350	8.54%
Low-income	77,229	0.41%	3,219	0.29%

*FOAM*, Free Open Access Medical education.

* Income level grouped by World Bank classification, 2016.
